# The role of different methanogen groups evaluated by Real-Time qPCR as high-efficiency bioindicators of wet anaerobic co-digestion of organic waste

**DOI:** 10.1186/2191-0855-1-28

**Published:** 2011-10-07

**Authors:** Deborah Traversi, Silvia Villa, Marco Acri, Biancamaria Pietrangeli, Raffaella Degan, Giorgio Gilli

**Affiliations:** 1 Department of Public Health and Microbiology, University of the Study of Turin, via Santena 5 bis, 10126, Turin, Italy; 2 SMAT S.p.A., corso XI Febbraio 14, 10152, Turin, Italy; 3 ISPESL, via Urbana 167, 00184, Rome, Italy

**Keywords:** methanogen, anaerobic digestion, biogas production, *Methanosarcina*, Archaea communities

## Abstract

Methanogen populations and their domains are poorly understood; however, in recent years, research on this topic has emerged. The relevance of this field has also been enhanced by the growing economic interest in methanogen skills, particularly the production of methane from organic substrates. Management attention turned to anaerobic wastes digestion because the volume and environmental impact reductions. Methanogenesis is the biochemically limiting step of the process and the industrially interesting phase because it connects to the amount of biogas production. For this reason, several studies have evaluated the structure of methanogen communities during this process. Currently, it is clear that the methanogen load and diversity depend on the feeding characteristics and the process conditions, but not much data is available. In this study, we apply a Real-Time Polymerase Chain Reaction (RT-PCR) method based on *mcrA *target to evaluate, by specific probes, some subgroups of methanogens during the mesophilic anaerobic digestion process fed wastewater sludge and organic fraction of the municipal solid waste with two different pre-treatments. The obtained data showed the prevalence of *Methanomicrobiales *and significantly positive correlation between *Methanosarcina *and *Methanosaetae *and the biogas production rate (0.744 p < 0.01 and 0.641 p < 0.05). *Methanosarcina *detected levels are different during the process after the two pre-treatment of the input materials (T-test p < 0.05). Moreover, a role as diagnostic tool could be suggested in digestion optimisation.

## Introduction

Methanogenesis is a characteristic unique to the Archaea (Woese 2007). Biological methane production involves 25 genes and numerous specific proteins and coenzymes. However, the gene number involved in the different aspects of methane production is much higher (Galagan et al. 2002). Methane can be produced through different pathways, each of which has a different substrate. Among the precursor organic molecules, we find CO_2_, formate, acetate and methyl groups. The CO_2_, with H_2 _as an electron donor, is reduced to methane via the hydrogenotrophic mechanism. Acetate is involved in the aceticlastic pathway, and the methyl group acts as the starting point of the methylotrophic pathway (Ferry 2010a, b). Anaerobic digestors are one typical habitat, especially for the following genera: *Methanobacterium, Methanothermobacter, Methanomicrobium, Methanoculleus, Methanofollis, Methanospirillum, Methanocorpusculum, Methanosarcina *and *Methanosaeta *(Liu and Whitman 2008). Two genera of Archaea, *Methanosarcina *and *Methanosaeta*, are methane producing from acetate, and this acetoclastic mechanism produces higher proportions of biogenic methane. These two genera are also the most studied in recent years with the advent of the complete genome sequencing of some strains (Barber et al. 2011). Methanogenesis is the final step of the anaerobic digestion process in the reactor. Other microorganisms, such as hydrolytic acidogens and acetogens, are involved in the previous steps. These microorganisms prepare the substrates for methanogenesis, which is considered to be the rate-limiting step (Rozzi and Remigi 2004). Anaerobic digestion technologies vary throughout Europe. For example, Germany has more than 4000 digesters (Dolan et al. 2011) and there are numerous examples of integrated management of waste and biomethane fuel production to provide public transport in Sweden and France (Lantz et al. 2007; Dolan et al. 2011). Recently, other countries have begun promotional projects to encourage anaerobic digestion methodology (Dolan et al. 2011). In Italy, the number of anaerobic digestion reactors is growing rapidly, especially farm-scale digesters (De Baere 2006). The fermentation of other organic waste is also financially appraised (Schievano et al. 2009a; Schievano et al. 2009b) in urban aggregation, where organic waste, such as the organic fraction of municipal solid organic waste (OFMSW) and wastewater sludge, are produced (Tambone et al. 2009; Pognani et al. 2009). To optimize the digestion benefits in terms of biogas production, waste volume reduction and waste impact on the environment, many research projects have begun in the past 10 years (Mata-Alvarez et al. 2011). The main results concern the parameters controlling the anaerobic process in technology configurations (Amani et al. 2010; Boe et al. 2010). Moreover, with recent technological and financial achievements, the microbiological aspects of anaerobic digestion have become relevant topics (Weiss et al. 2008; Cardinali-Rezende et al. 2009). This attention has led to the optimization of this process, which has paid for itself. Among the many microorganisms present in the reactor, methanogens are the most sensitive; however, they are difficult to study in culture-based methods, despite their critical role (Liu and Whitman 2008). In recent years, culture-independent techniques have been developed (Sekiguchi et al. 1998). These techniques are based on phylogenetic markers such as the 16S rRNA or methyl coenzyme M reductase (Mcr) genes (Nunoura et al. 2008; Rastogi et al. 2008). The 16S rRNA gene is the most widely used target for gene surveys (Nayak et al. 2009), whereas the Mcr is exclusive to the methanogens, with the exception of the methane-oxidising Archaea (Knittel and Boetius 2009; Whitman et al. 2006). The primary aim of this work is to study methanogen populations in order to find a bioindicator of a productive digestion process. To achieve this purpose, we determined, during anaerobic co-digestions, the abundance of methanogen subgroups utilising Real-Time qualitative PCR (RT-qPCR) with specific probes targeting the *mcrA *gene (additional file 1).

## Materials and methods

**Figure 1:**
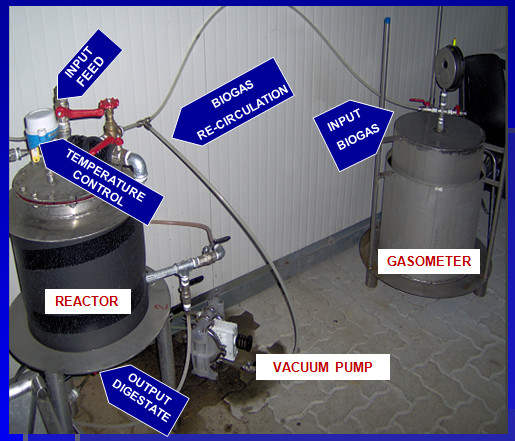
**The pilot hardware description is illustrated**. The same reactor, in different six-month fermentation sessions, with two different pre-treated feedings was used during this research study.

Two pilot reactors were fed pre-treated organic fractions of municipal solid waste (OFMSW) and wastewater sludge. The pre-treated methods used in this study included a pressure-extrusion (A) and a turbo mixing (B) system. In method A, the separation was achieved through a specially designed extruder press (280 bar) that separated the input waste into two fractions: a dry one to be sent to thermal conversion and a semi-solid one. The pressure-extruded dry fraction of the OFMSW was then diluted with wastewater sludge. By contrast, method B (the turbo-mixing system) was a wet process that works with a total solids (TS) content lower than 8%. The mixing and treating actions are performed by a rotating plate with hummers placed at the bottom of the turbo-mixing chamber that, when rotating at high velocity, induce the suspension to shear and crush. The particles weighing more than water precipitate to the bottom, where they are picked up by a screw and collected in an external vessel. The organic fraction remains in suspension and is pumped into a storage basin after passing through a shredding pump. In this case, OFMSW was directly turbo-mixed with wastewater sludge (about 1:3 proportion). The main physical-chemical characteristics of each kind of feed used in this work, just before entrance into the reactor, are shown in Table 1. The anaerobic co-digestion tests were conducted using a reactor with a total volume capacity of 15 L and a working volume of 10 L (Figure 1). The temperature was mesophilic and maintained at 38 ± 2°C using a water recirculation system connected to a thermostatic valve. The biogas produced was collected and measured in a calibrated gasometer and a mixing system containing the recirculated biogas produced during the anaerobic digestion process. The reactors were equipped with two openings, one at the top for feeding and one below to collect effluent discharge, as showed on Figure 1. Every day, 500 ml of digestate was removed from each reactor before adding another 500 ml of fresh feed. The parameters analysed three times a week in accordance with standard methods (APHA, 1995) included pH, total solids (TS), total volatile solids (TVS), alkalinity, acidity, nitrogen (N), and total carbon. Daily biogas production was measured using a liquid displacement system that was connected to the digester. The biogas volume was corrected using standard temperature and pressure conditions. The biogas composition (in terms of methane and carbon dioxide percentage) was analysed once a week with a portable analyser and confirmed by gas chromatography analysis.

**Table 1:** Characteristics of the pretreated inputs with the two different method used in the anaerobic co-digestion processes

	Pre-treatment A	Pre-treatment B
**pH**	4.4 ± 0.3	6.0 ± 0.7

**TS (%)**	9.9 ± 0.7	4.6 ± 1.1

**TVS (%)**	8.7 ± 0.7	3.3 ± 1.1

**TSV/TS (%)**	86.8 ± 0.2	70.6 ± 4.9

**C (%TS)**	46.0 ± 0.9	37.0 ± 3.4

**N (%TS)**	3.1 ± 0.2	3.5 ± 0.3

**C/N**	15.2 ± 1.1	10.4 ± 1.5

The reactors were operated at a constant organic loading rate of 4,5 ± 0,3 kg TVS/m^3 ^per day when OFMSW pressure-extruded was used and at an average organic loading rate of 1,7 ± 0,5 kg TVS/m^3 ^per day when OFMSW with pulper pretreatment was used. The tests were run over two consecutive hydraulic retention times of 20 days for each organic loading rate: one to ensure the highest replacement parts of the material inside the reactors and the other to analyse the process in a stable condition once all the feed had replaced the inoculum content. The main control parameters for pretreatments A and B are displayed in Table 2. Methanogen subgroups were determined using samples with the highest biogas production rate. These included 15 from pretreatment A and 10 from pretreatment B. The samples were collected during 2009 in 50 ml sterile tube and frozen at -20°C until the extraction session.

**Table 2:** Main relevant evaluation parameters of the co-digestion processes divided by pre-treatment method

Parameters	Pre-treatment A	Pre-treatment B
Daily biogas production (L/die)	27.08 ± 3.01	4.87 ± 2.46

Specific Biogas production (m^3^/kg VS _added_)	0.64 ± 0.07	0.30 ± 0.13

TS reduction (%)	64.44 ± 7.57	31.67 ± 6.23

TSV reduction (%)	73.84 ± 5.87	38.13 ± 6.70

pH	7.36 ± 0.34	6.82 ± 0.52

Ac./Alc. ratio	0.37 ± 0.18	2.47 ± 2.41

CH_4 _(%)	60.60 ± 2.90	57.50 ± 6.10

CO_2 _(%)	37.70 ± 3.20	41.00 ± 6.44

### DNA extraction and purification

The digestate aliquots were thawed at 4°C overnight and centrifuged at 4000 g for 10 minutes. After removing the supernatant, semi-dry aliquots were used for the following steps. Total DNA was extracted from 0.25 g of this particulate matter (residue humidity was equal to 31 ± 5%) using the PowerSoil DNA Isolation Kit following by UltraClean Soil DNA Kit (MoBio Laboratories). The average DNA quantity extracted was 3.51 ± 1.53 ng/μl, and DNA quality was evaluated by gel electrophoresis before the chain reaction. Only samples with a DNA quantity above 1 ng/μl and of sufficient quality were used for the following step.

### qRT-PCR analysis

After DNA extraction and purification, different methanogens were quantified using methanogen-specific short primers for a *mcrA *sequence (Steinberg and Regan 2008) and synthesised by ThermoBiopolymer and previously described specific probes (Steinberg and Regan 2009).

*Methanosarcina, Methanobacterium, Methanocorpusculum *and *Methanosaeta *were determined with the respective following probes: *msar, mrtA, mcp *and *msa *(Steinberg and Regan 2009). The reactions were conducted in singleplex with a standard super mix (Bio-Rad iQ™ Multiplex Powermix) using RT-PCR Chromo4 (Bio-Rad) and Opticon Monitor 3 Software. The reaction conditions have been previously described (Steinberg and Regan 2009, 2008).

Standard references were available only for the *Methanosarcina *and *Methanobacterium*. The references were a *Methanosarcina acetivorans mcrA *sequence and a *Methanobacterium thermoautotrophicum mrtA *sequence. Each plasmid is included in pCR21 vector (Invitrogen) supplied by L.M. Steinberg and J.M. Regan, Pennsylvania State University. These plasmids were amplified, transforming *Escherichia coli *Top10 cells according to the manufacturer's instructions. Transformed cells were selected on LB agar with ampicillin, and the plasmid was extracted using a plasmid DNA purification kit (NucleoSpin Plasmid, Macherey-Nagel). The standard curve had six points, and it was calculated using the threshold cycle method with the highest standard amplified being 2.3 ng of plasmid (~4.5*10^8 ^plasmid copies). Between each following standard curve point, there is a 1:10 dilution. Standards and samples were tested in triplicates. The triplicate averages were accepted only if the coefficient of variation was below 20%. Example of regression curves with correlation coefficient and PCR efficiency were showed on Table 3. Resolution limit of the method was settled to 4.5*10^3 ^copies of *mcrA*. The PCR products are about 500 base pairs long.

**Table 3:** qRT-PCR probe and reaction descriptions

Target group	Probe name target	Example of regression curve	**r**^**2**^	PCR efficiency (%)	Acceptable data (%)
Methanosarcina	msar	y = -0.2547x +11.34	0.997	**80**	75

Methanobacteriaceae	mrtA	y = -0.2691x+12.21	0.995	86	4

Methanocorpusculaceae	mcp	y = -0.2627x+12.38	0.987	83	88

Methanosaetaceae	msa	y = -0.2380x+10.27	0.943	73	52

There is a standard reference curve only for the *Methanosarcina *and *Methanobatecteriaceae*, making it possible to establish the gene copies in the extracted DNA. The last column indicates the percentage of determinable sample on the total 25 tested samples.

For *Methanocorpusculaceae *and *Methanosaetaceae*, there was no standard reference available; therefore, quantification could only be considered between samples in the same analytical session. The efficiency of the PCR reactions was determined with serial 1:10 dilution of a sample and are showed on Table 3. The results for these groups were expressed as cycle threshold (Ct) or as 1/Ct, where relative abundance was discussed for each reaction, instead of real quantification, as for the *Methanosarcinaeae and Methanobacterium*, where results could be expressed as gene copies per microliter of DNA extract.

We used 2 μl of a 1:5 dilution of DNA extracts for amplification. This quantity of sample was evaluated as the best among various tested quantities for obtaining quantifications within the standard curve range and with acceptable PCR efficiency. The 1:5 dilution is sufficient to avoid the effect of inhibition substances present in this kind of sample. Only a percentage of the 25 total samples were acceptable as detailed on the table 3, and values ranged by methanogen group from 4 to 88. In many samples, evaluation of the Ct was not determinable (above 40).

To evaluate precision, we began with the same two samples re-extracted 10-fold. The results of the successive PCR-determination showed a variation coefficient below 6% for *msar *amplification and below 15% for *msa, mrtA *and *mcp *amplifications.

### Statistics

Statistical analyses were performed using the SPSS Package, version 17.0, for Windows. A Spearman correlation coefficient was used to assess the relationships between variables. A T-test of independent variables was used to test mean evaluations. The differences and correlations were considered significant at p < 0.05 and highly significant at p < 0.01.

## Results

The detected level of various methanogen groups is displayed in Table 4. Groups varied largely in quantity during the digestion processes and were often not present at all. *Methanosarcina *was not detected in some samples, this happened when the pH was around 6.5 and the production rate was lower than 0.5 m^3^/kg VS_added_. The number of *msar *copies in the sample can be explained by the relevant level of acetate, the substrate of this group, and the high biogas production rate recorded from the reactor. As described in the literature, an anaerobic digester typically contains more than 10^12 ^cells/μl with an average of 10^8 ^methanogens (Amani et al. 2010). *Methanobacteriaceae mrtA *resulted undetectable nearly in all the samples (table 3) while the *Methanomicrobiales *resulted prevalent, in particular acetoclastic methanogens (*Methanosarcin*a and *Methanosaeta*). Furthermore, their presence increased along with the specific biogas production rate (Table 5). *Methanocorpusculaceae *seemed to have a similar behaviour as showed in table 5 and their presence is highly correlated both to *Methanosarcina *and *Methanosaeta*. *Methanosarcina *was significantly correlated with all the control parameters (positively with the pH, specific biogas production and % TSV; negatively with the acidity/alkalinity ratio) as showed on table 4. With increases in the TVS, there was also an increase in *Methanocorpusculaceae *and *Methanosaetaceae*. A significant, positive correlation with the pH was also observed for the other acetoclastic group, *Methanosaetaceae *(Table 4).

**Table 4:** Descriptive analysis of the acceptable data by each probe

Target (measure unit)	Min	Max	Mean	**Dev. std**.
Methanosarcina (gene copies/μl)	4.77E+04	6.03E+07	1.19E+07	1.51E+07

Methanobacteriaceae (gene copies/μl)	1.52E+05	1.52E+05	1.52E+05	-

Methanocorpusculaceae (1/Ct)	2.52E-02	3.98E-02	2.966E-02	3.6E-03

Methanosaetaceae (1/Ct)	2.56E-02	3.74E-02	2.969E-02	3.7E-03

**Table 5:** Spearman's rho correlation between the detected methanogen groups and the monitored control parameters

	pH	Ac/Alc ratio	% TVS added	**Biogas production (m**^**3**^**/kg VS **_**added**_**)**	msar (gene copies/μl)	msa (1/Ct)
msar (gene copies/μl)	0.630**	-0.589**	0.744**	0.673**	1	0.782**

msa (1/Ct)	0.847**	-	0.641*	0.576*	0.782**	1

mcp (1/Ct)	-	-	0.449*	-	0.719**	0.868**

Significant correlation at p < 0.05 is identified with a single asterisk while highly significant at p < 0.01 with a double asterisk. The hyphen is introduced when no significant correlations (n.c.) were observed.

**Figure 2:**
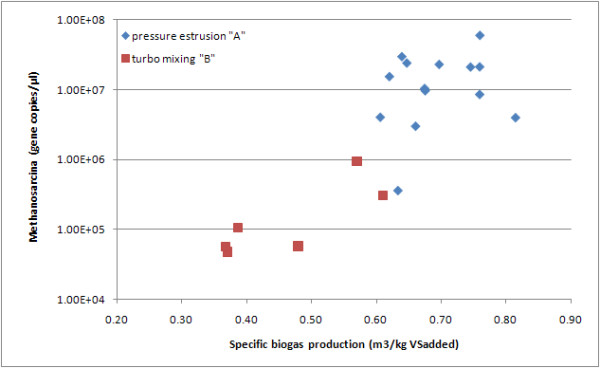
**The quantification of *Methanosarcina *during the two monitored processes in relation to specific biogas production rate subdivided by pre-treatment**.

The significant correlations among the various methanogen groups and control parameters are displayed on Table 5. In Figure 2, the *Methanosarcina *loads were differentiated in relation to the pre-treatment of the input material (A and B). The difference between the mean of the *Methanosarcina *levels, during the digestion with the pressure-extrusion input, is significantly higher than the turbo-mixing one (1.68E7 *vs *2.55E5, F = 6.821, p = 0.018).

Moreover the figure 2 illustrates as all the samples, collected during the process conducing after pressure-estrusion pre-treatment, showed a biogas production rate above or near to 0.6 m^3^/kg TSV_added_. This cut-off is a suitable division between optimal and suboptimal digestion conditions as has been documented in the literature (Amani et al. 2010).

## Discussion

Anaerobic digestion is among the most complicated and unknown biological processes in the environment (Schink 1997). Different aspects attract operational, chemical and biological criticisms. Moreover, these aspects are strictly interconnected with one another. A wide number of papers in this field have been published in recent years (Khalid et al. 2011). Most of these studies, however, didn't include methanogens characterization or they have been based on a metagenomic approach in which a small subunit of ribosomal RNA was used (Pycke et al. 2011; Supaphol et al. 2011). Methanogen studies using the *mcrA*-based method have become more common in recent years (Narihiro and Sekiguchi 2011).

Over 90% of the detected methanogenic Archaea in the mesophilic reactor fed swine slurry belonged to the hydrogenotrophic methanogens. These were predominantly *Methanobacteriales *followed by *Methanomicrobiales *(Zhu et al. 2011). On the other hands always in mesophilic biogas plant but fed with cattle manure, 84% of all detected methanogens were affiliated with the *Methanomicrobiales*, whereas only 14% belonged to the *Methanosarcinales *and 2% to the *Methanobacteriales *(Bergmann et al. 2010a, b) and in other plant always running on cattle manure, the methanogen community presented the following composition: 41.7% of clones were affiliated with *Methanomicrobiales*, 30% with *Methanosarcinales*, and 19% with *Methanobacteriales; *at temperatures lower than 25°C, the *Methanomicrobiales *became most prevalent (> 90%) (Rastogi et al. 2008).

In reactor fed leachate and OFMSW, various orders of hydrogenotrophic methanogens belonging to *Methanomicrobiales *and *Methanobacteriales *were identified (Cardinali-Rezende et al. 2009). However, during mesophilic digestion of wastewater sludge, *Methanosarcina *and *Methanosaeta *were most abundant, comprising up to 90% of the total Archaea present or more (Narihiro et al. 2009; Das et al. 2011). This data confirms the results of our work and the ability of *Methanosarcina *species to form multicellular aggregates that may resist inhibitions in the reactor (Vavilin et al. 2008).

Despite the data variability such bio-molecular approach can improve the available knowledge of anaerobic digestion, as demonstrated in this work, the biogas production efficiency is significantly and positively correlated to two methanogen groups (*Methanosarcina *and *Methanosaetaceae*). Most importantly, this method can represent a way to introduce useful bioindicators into the reactors for early diagnosis of an unbalance or a sufferance situation in the microbiologic community. Establishing an efficiency cut-off during the anaerobic digestion process - optimal production that for our set up is around 0.6 CH_4 _m^3^/kg SV_added _- it makes possible to observe a role for certain groups of methanogens, primarily the *Methanosarcina *as useful Archaea bioindicators in the digestion process. On the other hands the produced data shows a clear advantage in the pressure-extrusion respect to turbo-mixing pre-treatment as production rate moreover also the cost of the two pre-treatment plants are very different, against the pressure-extrusion. After a validation process with different digestion processes, the definition of a threshold of alarm seems to be possible.

Finally, it is critical that this kind of approach be utilised and that knowledge in this scientific field be increased. The methanogen diversity in the reactor is widely influenced by the feeding. During anaerobic digestion in which input is mainly cattle manure, the presence of hydrogenotroph methanogens is favoured. However, when other feedings are involved, as in this experimental activity, the methanogen community structure differs in terms of the prevalence of *Methanosarcineae *such as *Methanosarcina *and *Methanosaeta*. This family presents a prevalent acetoclastic methane production. A closer examination is needed for substrate and product analysis. A profile of the substrates, such as butyrate, propionate, H_2 _and CO_2_, could be useful in understanding the microbiologic dynamics and the consequent methanogen modulations.

## Competing interests

The authors declare that they have no competing interests.

## Supplementary Material

Additional file 1 **Graphical abstract**. During mesophilic anaerobic co-digestion, biomolecular methanogen determinants in the reactor vary among groups in different biochemical pathways, indicating that variation in biogas yield supplies early bioindicators of methane production. Click here for file
