# Supplementary figures and images for: The role of different methanogen groups evaluated by Real-Time qPCR as high-efficiency bioindicators of wet anaerobic co-digestion of organic waste
Source: AMB Express. 2011 Oct 7;1:28. doi: 10.1186/2191-0855-1-28 (PMC3219682; doi:10.1186/2191-0855-1-28)

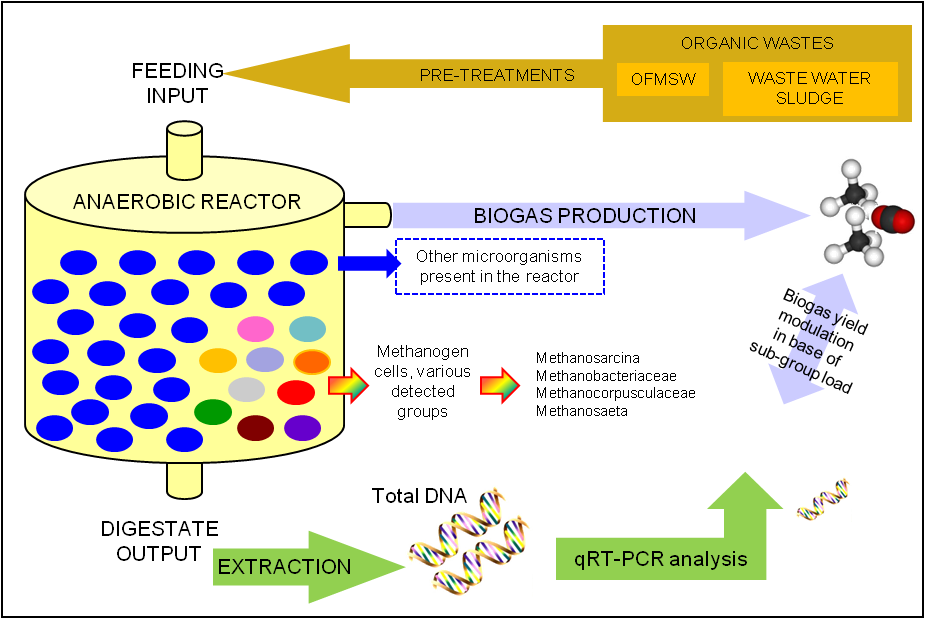

Supplement: Additional file 1 — Graphical abstract. During mesophilic anaerobic co-digestion, biomolecular methanogen determinants in the reactor vary among groups in different biochemical pathways, indicating that variation in biogas yield supplies early bioindicators of methane production. [file 2191-0855-1-28-S1.TIFF]
